# Impact of Gastric *H*^+^*/K*^+^*-ATPase* rs2733743 on the Intragastric pH-Values of Dexlansoprazole Injection in Chinese Subjects

**DOI:** 10.3389/fphar.2017.00670

**Published:** 2017-09-22

**Authors:** Lu-Ning Sun, Yang Cao, Yue-Qi Li, Yun-Qian Fang, Hong-Wen Zhang, Mei-Feng Wang, Li-Jun Xie, Juan Chen, Zhi-Cheng Yang, Ming-Liang Bian, Hao Li, Pei-Pei Zhang, Ji-Fu Wei, Ling Meng, Xue-Hui Zhang, Ping Zhao, Yong-Qing Wang

**Affiliations:** ^1^Research Division of Clinical Pharmacology, First Affiliated Hospital with Nanjing Medical University Nanjing, China; ^2^Department of Gastroenterology, First Affiliated Hospital with Nanjing Medical University Nanjing, China; ^3^Department of Pharmacy, Jiangsu Shengze Hospital Suzhou, China

**Keywords:** gastric *H*^+^*/K*^+^*-ATPase*, gastric acid, pharmacokinetics, pharmacogenomics, *CYP2C19*

## Abstract

**Background:** Not all patients with acid-related disorders receiving proton pump inhibitor (PP) treatment get adequate gastric pH control. The genetic variation of receptors, metabolic enzymes, and transporters are known to cause failures of therapies. We have conducted a study to evaluate the influence of gastric *H*^+^*/K*^+^*-ATPase, CYP2C19*, and *ABCB1* polymorphisms on the pharmacokinetic and pharmacodynamic profiles of dexlansoprazole injection in healthy Chinese subjects.

**Methods:** A total of 51 subjects were enrolled for pharmacokinetic and pharmacodynamic study after a single intravenous administration of 20 or 30 mg dexlansoprazole. Plasma concentrations were determined using a chiral liquid chromatography-mass spectrometry method. The intragastric pH and baseline-adjusted intragastric pH parameters were introduced to evaluate the pharmacodynamic characters. Genotyping was performed by polymerase chain reaction.

**Results:** The pharmacokinetic parameters were significantly influenced by *CYP2C19* phenotypes, and gastric acid secretion inhibition were affected by both gastric *H*^+^*/K*^+^*-ATPase* and *CYP2C19* polymorphisms. Gastric H^+^*/K*^+^-ATPase genotypes had greater effects than *CYP2C19* genotypes on the suppression of gastric acid secretion.

**Conclusion:** Gastric H^+^*/K*^+^-ATPase polymorphism may be one of the main reasons that cause insufficient gastric acid inhibition.

## Introduction

Gastric hydrogen-potassium adenosine triphosphatase (H^+^/K^+^-ATPase, proton pump), an α,β-heterodimeric enzyme, is responsible for the final step of gastric acid production (Abe et al., 2011). It serves as the target of proton pump inhibitors (PPIs) for the treatment of gastric acid-related diseases including peptic ulcer disease, gastro-esophageal reflux disease (GERD), erosive esophagitis, and acute upper gastrointestinal hemorrhage (Mullin et al., 2009; Gralnek et al., 2015). Approximately 20% of people in the western population experience symptoms of GERD regularly (Hershcovici et al., 2011), and the prevalence of peptic ulcer disease is over 16% in Chinese population (Lau et al., 2013). However, the acid-suppressive effect of PPIs varies between individuals, for instance, about 19% of GERD patients experience persistent symptoms after standard doses of PPIs therapy due to inadequate acid inhibition (Katz, 2006).

The inhibition of gastric acid secretion caused by PPIs depends on their covalent binding to gastric H^+^/K^+^-ATPase (Sachs et al., 2007). The α-subunit of this enzyme, constituted of 1,034 amino acids, has 10 transmembrane segments (TMs) (Shin and Sachs, 2004, 2006). Besides one common site (cysteine 813 at exoplasmic vestibule entry into TM6) that binds with all PPIs, omeprazole binds to cysteine 892, lansoprazole binds to cysteines 892 and 321, pantoprazole binds to cysteine 822 and rabeprazole binds to cysteines 892, 822, and 321(Besancon et al., 1997; Shin and Sachs, 2004, 2006). The affinity of inhibitors is decreased when amino acids mutate at the extra cellular end of TM6, the loop between TM5 and TM6, or the luminal end of TM8 (Sachs et al., 2007), which would lead to lower acid secretion activity. Single nucleotide polymorphisms (SNPs) of the α-subunit may have a correlation with the configuration of gastric H^+^/K^+^-ATPase, which may lead to differences in individual gastric acid inhibition by PPIs (Li et al., 2013). However, the relation between SNPs of *H*^+^*/K*^+^*-ATPase* and the inhibition of acid secretion by PPIs in humans has not been investigated.

Besides the binding between PPIs and proton pump, the pharmacological response of acid inhibition is also related to the systemic exposure of drugs (Vakily et al., 2009). PPIs are extensively metabolized by cytochrome P450 (CYP) 2C19 (Mullin et al., 2009). Our previous studies showed that there were significant relationships between *CYP2C19* polymorphisms and pharmacokinetics or pharmacodynamics after healthy volunteers were administered omeprazole (Wang et al., 2010; Feng et al., 2015), rabeprazole (Wang et al., 2011), lansoprazole (Wang et al., 2012), or pantoprazole (Gawrońska-Szklarz et al., 2012). Lansoprazole is a substrate of ABCB1 (ATP-binding cassette, sub-family B, member 1) protein, which pumps xenobiotics (such as drugs) out of cells (Aller et al., 2009). The pharmacokinetic (PK) and pharmacodynamic (PD) difference between wild-type and mutant types of *ABCB1 C3435T* after an oral administration of lansoprazole is inconsistent (Kodaira et al., 2009; Li et al., 2014). To date, the influence of *CYP2C19* and *ABCB1* genetic polymorphisms on PK and PD of dexlansoprazole have not been reported.

Being the R-enantiomer of lansoprazole, dexlansoprazole has a lower clearance and a higher systemic exposure than the S-enantiomer, which could provide improved PK profiles in humans (Katsuki et al., 1996; Metz et al., 2009; Sun et al., 2015). The currently marketed formulation of dexlansoprazole is a dual delayed-release (DDR) capsule indicated for erosive esophagitis and GERD, which was first approved by the FDA in 2009. The novel formulation for injection was developed for the treatment of acute upper gastrointestinal hemorrhage by providing consistently high gastric pH (Gisbert et al., 2001).

The aim of this study is to evaluate the influence of gastric *H*^+^*/K*^+^*-ATPase, CYP2C19*, and *ABCB1* polymorphisms on the gastric acid inhibition and pharmacokinetics profiles of dexlansoprazole injection in healthy Chinese subjects.

## Materials and methods

### Study design

This study was an open-label and single-center clinical trial (China Food and Drug Administration registration: 2013L01977). The protocol was approved by the Ethics Committee of First Affiliated Hospital with Nanjing Medical University and was conducted in accordance with the Declaration of Helsinki and Good Clinical Practice guideline. All participants gave written informed consent prior to the enrollment.

### Subjects

A total of 328 subjects were enrolled for the genetic analysis of gastric *H*^+^*/K*^+^*-ATPase* polymorphisms. Among this pharmacogenetic population, 51 subjects participating in the PK and PD study were sampled for genotyping of *CYP2C19* and *ABCB1* additionally.

Eligible subjects for PK and PD study were selected from healthy Chinese subjects aged 18–40 years, with a body mass index of 19–24 kg/m^2^. Subjects were examined to be healthy on the basis of medical history, physical examination, laboratory examination, and 12-lead electrocardiogram. The following exclusion criteria were applied to subjects in PK and PD study: a history of clinically significant cardiovascular, hepatic, renal or gastrointestinal diseases; a history of nervous system or muscle disease; seizure or other psychiatric disorders; a history of known allergy or intolerance to any drugs; a history of tobacco, alcohol, or drug abuse; a positive outcome of *Helicobacter pylori* infection test; those with abnormalities in clinical laboratory parameters; reception of an experimental drug or donation of blood 3 months prior to the first dose; pregnant or nursing female.

All subjects were confined to the Phase I unit beginning with the evening before baseline intubation and kept to a regular schedule. An overnight fasting (12 h) was required before administration, while standard meals were provided 4 h post-dose.

### Study drug and administration

Dexlansoprazole injection (30 mg per vial) used in the PK and PD study was manufactured by Nanjing Yoko Pharma Co., Ltd. (Nanjing, China). Subjects received an intravenous infusion of 20 or 30 mg dexlansoprazole injection in sterile saline solution (100 mL/h, 60 min). The study drug was administrated with an infusing pump (SA213, Lifepum, Beijing, China) and given through an intravenous catheter in the antecubital vein opposite to the one from which blood samples were taken.

### Pharmacokinetic measurement

To determine the pharmacokinetic properties of dexlansoprazole injection in human, a series of venous blood samples (5 mL) were collected in heparinized tubes at 0 (pre-dose), 10, 20, 40, 60, and 75 min, 1.5, 2, 2.5, 3, 4, 6, 9, 12, 16, 24, and 36 h post-dose. Samples were centrifuged immediately at 4°C for 10 min at 3,500 revolutions per minute, then separated and stored at −80°C for PK analysis.

A chiral liquid chromatography-mass spectrometry method was developed and validated for the simultaneous determination of (R)- and (S)-lansoprazole in human plasma, with the calibration curve of 500–3,000 ng/mL for both (R)- and (S)-lansoprazole (Sun et al., 2015). Baseline chiral separation was achieved on a Chiralpak IC column. The mass spectrometric analysis was performed using a QTrap 5500 mass spectrometer (AB Sciex, Concord, Ontario, Canada).

The PK parameter of C_max_ (maximum concentration) and T_max_ (the time to C_max_) were obtained directly from the observed data. Other PK parameters including *t*_1/2_ (elimination half-life), AUC^PK^ (area under the plasma concentration-time curve), V_d_ (volume of distribution), and CL (clearance) were calculated using non-compartmental methods with Phoenix WinNonlin version 6.4 (Pharsight Inc., CA, USA).

### Pharmacodynamic measurement

An ambulatory pH recorder (Orion II, MMS, USA) with a disposable pH catheter (VersaFlex, MMS, USA) was used to monitor intragastric pH. The electrode was placed in water for at least 10 min and then calibrated using standard buffer solutions (pH 1.07 and 7.01) before intubation. Both calibration and intubation processes were conducted under the guidance of MMS Virtual Instructor Program. The catheter, trans nasally placed 10 cm under the lower esophageal sphincter, was identified by a sharp decrease in pH (Wang et al., 2010, 2011). Subjects were intubated on baseline and the day of dosing at about 7:30 a.m., and the intragastric pH data were recorded continuously for 24 h from 8:00 a.m. Intragastric pH was sampled every second, and the data were stored in a MMS Database program (MMS, USA). The PD parameters including Median (pH), Mean (pH), T_pH≥3_ (%), T_pH≥4_ (%), T_pH≥6_ (%), T_pH≥6_, and AUC^pH−t^ were analyzed by the MMS Database program.

Median (pH) and mean (pH) were well-investigated in most studies, and the percentage time of pH ≥ 4 (or pH ≥ 4 holding time ratio) was the benchmark for predicting clinical efficacy of PPIs in treating acid-related diseases (Metz et al., 2009). Novel parameter T_pH≥6_ (time to first gastric pH ≥ 6, min) and ${\text{AUC}}_{\tau \;1-\tau \;2}^{\text{pH}-\text{t}}$ (area under pH-time curve from τ1 to τ2, pH·s) were defined to evaluate the velocity and extent of pharmacodynamics with more accuracy. All parameters were calculated and compared according to time periods of 0–24, 0–4, 4–10, 10–14, and 14–24 h post dosing.

The parameter ${\text{AUC}}_{\tau \;1-\tau \;2}^{\text{pH}-\text{t}}$ was calculated as follows:

$$\begin{array}{l} {\text{AUC}}_{\tau \text{1}-\tau \text{2}}^{\text{pH}-\text{t}}=10\times \text{t}-{\text{AUC}}_{\tau \text{1}-\tau \text{2}}^{\text{10}-\text{t}} \end{array}$$

Where *t* represented the time duration measured by seconds, ${\text{AUC}}_{\tau \;1-\tau \;2}^{10-\text{t}}$ was the area between the pH line = 10 and the pH-time curve, which was generated by the MMS software.

Baseline-adjusted PD parameters were calculated as follows: for example, ΔAUC^pH−t^ = AUC^pH−t^ − AUC^pH−tbaseline^, ΔMean (pH) = Mean (pH) − Mean (pH)^baseline^, and ΔMedian (pH) = Median (pH) − Median (pH)^baseline^.

### Gastric *H^+^/K^+^-ATPase, CYP2C19*, and *ABCB1* genotyping

Genomic DNA was extracted from leucocytes of blood samples using a commercially available kit (RelaxGene Blood DNA System, Tiangen, Beijing, China) according to the manufacturer's protocol. A total of 14 exons of *ATP4A* gene were sequenced using primers as Oksanen (Oksanen et al., 2006) published. Genotyping for *H*^+^*/K*^+^*-ATPase* (rs2733743), CYP2C19^*^2 (rs4244285), CYP2C19^*^3 (rs4986893), CYP2C19^*^17 (rs12248560), and *ABCB1 C3435T* (rs1045642) variant alleles was performed by polymerase chain reaction followed by Sanger sequencing. DNA sequencing was processed using BigDye® Terminator Cycle Sequencing v3.1 kit and DNA Analyzer (3730xl, Applied Biosystems, USA), which was provided by Realgene Biotech (Nanjing, China) and Sangon Biotech (Shanghai, China). Subjects participating in PK and PD studies were genotyped into three groups according to *CYP2C19* wild-type gene, ^*^2 and ^*^3, namely homEM (^*^1/^*^1), hetEM (^*^1/^*^2 and ^*^1/^*^3), and PM (^*^2/^*^2, ^*^2/^*^3, and ^*^3/^*^3).

### Statistical analysis

Descriptive statistics was utilized to summarize demographic and biologic characteristics of subjects. Continuous variables (PK and PD parameters) were expressed as Mean ± SD, and categorical variables as frequencies and percentages. Genotype frequencies were tested for Hardy-Weinberg equilibrium. Statistical comparison of PK and PD parameters was performed by an independent sample test and one-way analysis of variance (ANOVA) with two-sided *P* < 0.05 considered statistically significant. Statistical analysis was performed using SPSS software version 22.0 (IBM Corp., USA).

## Results

### Study population

Demographic and biologic variables are summarized in Table 1. Genotype and allele frequencies of gastric *H*^+^*/K*^+^*-ATPase* (rs2733743), CYP2C19^*^2 (rs4244285), and *ABCB1 C3435T* (rs1045642) were distributed according to the Hardy-Weinberg equilibrium in the study population. No CYP2C19^*^17 (rs12248560) mutant was observed in the PK and PD population. In the pharmacogenetic analysis of the gene encoding the gastric H^+^*/*K^+^-ATPase α-subunit, one non-synonymous variation (rs2733743) was found in most subjects with 115 heterozygotes (35.1%) and 123 homozygotes (37.5%). The recorded SNP rs2733743, a homozygous A → G transition (c.823T>C) in *ATP4A*, alters amino acid 265 of gastric H^+^*/*K^+^-ATPase α subunit from valine to alanine (Yang et al., 2012). Subjects were then classified into three types as gastric *H*^+^*/K*^+^*-ATPase* wildtype, heterozygote and homozygote (AA, AG, and GG, respectively) accordingly.

**Table 1:** Demographic and biologic characteristics.

**PG POPULATION (*****n*** = **328)**			
***H**^**+**^**/K**^**+**^**-ATPase*** **rs2733743 Genotype, Counts (%)**			
Wild-type (AA)			90 (27.4)
Heterozygote (AG)			115 (35.1)
Homozygote (GG)			123 (37.5)
**PK AND PD POPULATION (*****n*** = **51)**			
**Demographic Variable, Mean** ± **SD or Counts (%)**			
Race	Han, *n* (%)	45 (94.1)	
	Other, *n* (%)	3 (5.9)	
Age, years			25 ± 3
Height, m			1.67 ± 0.07
Weight, kg			60 ± 7
BMI, kg/m^2^			21.7 ± 1.2
***H**^+^**/K**^+^**-ATPase*** **rs2733743 Genotype, Counts (%)**			
Wild-type (AA)			14 (27.5)
Heterozygote (AG)			17 (33.3)
Homozygote (GG)			20 (39.2)
***CYP2C19*** **Genotype, Counts (%)**			
homEM (^*^1/^*^1)			19 (37.3)
hetEM (^*^1/^*^2, ^*^1/^*^3)			17 (33.3), 2 (3.9)
PM (^*^2/^*^2)			13 (25.5)
***ABCB1 C3435T*** **Genotype, Counts (%)**			
Wild-type (CC)			24 (47.1)
Heterozygote (CT)			19 (37.2)
Homozygote (TT)			8 (15.7)

*PG, pharmacogenetic; PK, pharmacokinetic; PD, pharmacodynamic; CYP2C19, cytochrome P450 2C19; ABCB1, ATP-binding cassette, sub-family B, member 1*.

### Pharmacokinetic data

**Figure 1:**
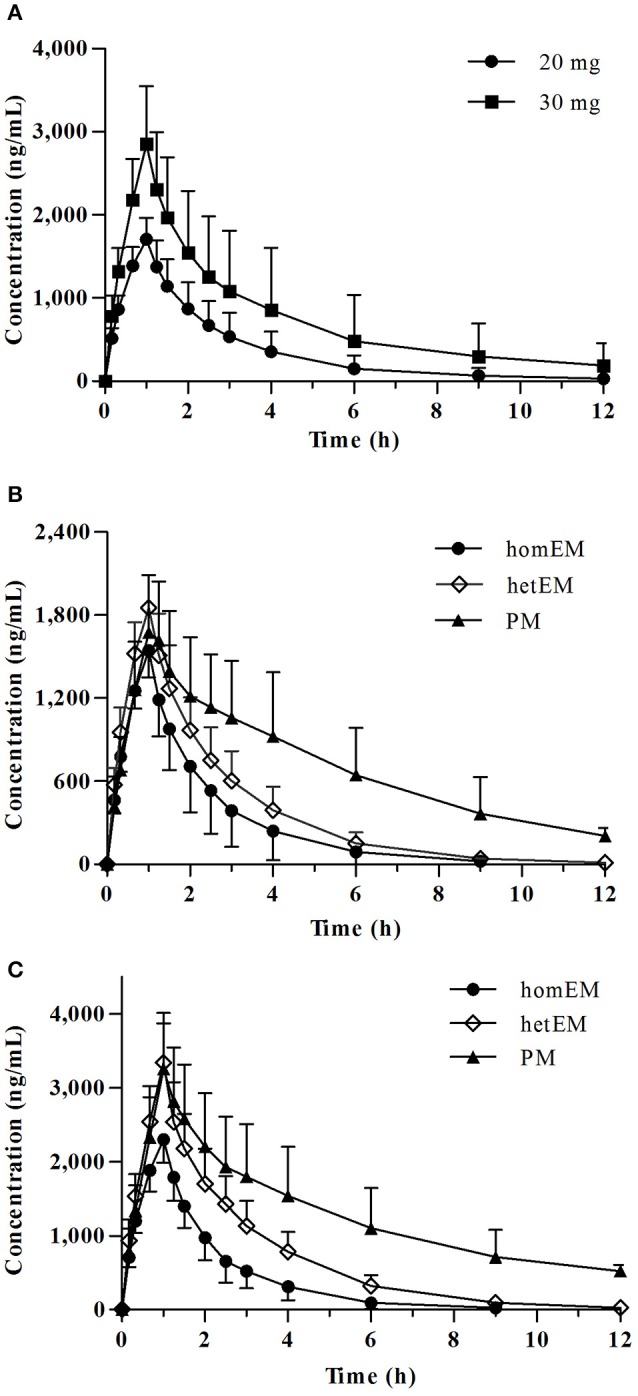
Plasma concentration-time profiles for dexlansoprazole 20 and 30 mg. **(A)** Mean ± SD concentration-time curve for dexlansoprazole after a single intravenous dosing of 20 vs. 30 mg. Comparison of plasma concentrations following intravenous administration of dexlansoprazole **(B)** 20 mg (*n* = 8, 9, 6 for homEM, hetEM, and PM) and **(C)** 30 mg (*n* = 11, 10, 7 for homEM, hetEM, and PM) by *CYP2C19* phenotype.

Mean plasma concentration-time profile of dexlansoprazole after single intravenous administration is shown in Figure 1A. The PK parameters for dexlansoprazole injection grouped by dose are summarized in Table 2. The C_max_ and AUC^PK^ exhibit dose proportionality with no statistical difference in ln (C_max_/dose) and ln (AUC^PK^/dose) between the 20 and 30 mg groups. The PK parameters of *t*_1/2_, CL, and V_d_ were independent of dosage, and no significant gender-related difference was observed in PK parameters except that the V_d_ of male subjects was slightly higher than that of female subjects.

**Table 2:** PK parameters for dexlansoprazole following a single intravenous administration of 20 or 30 mg by *CYP2C19* phenotype (Mean ± SD).

**Dosage (mg)**	***CYP2C19* genotype**	**C_max_ (ng/mL)**	**T_max_ (h)**	***t*_1/2_ (h)**	**${\text{AUC}}_{0-\text{t}}^{\text{Con}-\text{t}}$ (ng·h/mL)**	**${\text{AUC}}_{0-\infty }^{\text{Con}-\text{t}}$ (ng·h/mL)**	**V_d_ (L)**	**CL (L/h)**
*20*	homEM (*n* = 8)	1, 544 ± 193^b^	1.00 ± 0.00	1.18 ± 0.40	3, 365 ± 1, 538	3, 392 ± 1, 538	10.40 ± 1.07^b^	6.75 ± 2.27^b^
	hetEM (*n* = 9)	1, 853 ± 238^a^	1.00 ± 0.00	1.37 ± 0.24	4, 618 ± 1, 253	4, 643 ± 1, 261	8.78 ± 1.35^a^	4.60 ± 1.25^a^
	PM (*n* = 6)	1,673	1.00	3.65	9,488	9,600	10.98	2.08
	Total (*n* = 23)	1, 706 ± 257	1.00 ± 0.00	1.41 ± 0.64	4, 332 ± 1, 939	4, 362 ± 1, 955	9.62 ± 1.45	5.41 ± 2.17
*30*	homEM (*n* = 11)	2, 299 ± 307^b^^,^^c^	1.00 ± 0.00	1.14 ± 0.28^c^	4, 641 ± 1, 293^c^	4, 665 ± 1, 299^c^	10.71 ± 1.23^b^	6.89 ± 1.94^b^^,^^c^
	hetEM (*n* = 10)	3, 347 ± 669^a^	1.00 ± 0.00	1.56 ± 0.15^c^	8, 571 ± 2, 174^c^	8, 595 ± 2, 179^c^	8.20 ± 1.73^a^	3.69 ± 1.05^a^
	PM (*n* = 7)	3, 256 ± 619^a^	1.00 ± 0.00	3.96 ± 1.54^a^^,^^b^	18, 355 ± 7, 432^a^^,^^b^	18, 439 ± 7, 466^a^^,^^b^	9.58 ± 1.56	2.41 ± 2.43^a^
	Total (*n* = 28)	2, 851 ± 700	1.00 ± 0.00	2.17 ± 1.57	10, 086 ± 7, 510	10, 130 ± 7, 544	9.78 ± 1.69	4.69 ± 2.80

a P < 0.05 vs. CYP2C19 homEM;

b P < 0.05 vs. CYP2C19 hetEM;

c *P < 0.05 vs. CYP2C19 PM*.

*CYP2C19* PMs presented a slower elimination and higher exposure (C_max_ and AUC^PK^) compared to *CYP2C19* homEMs, while the elimination and exposure of *CYP2C19* hetEMs were intermediate between that of PMs and homEMs (Figures 1B,C). The PK parameters grouped by *CYP2C19* phenotype are summarized in Table 2. In the 30 mg group, *CYP2C19* PMs exhibit significantly higher C_max_, AUC^PK^, and *t*_1/2_ than homEMs and presented remarkably higher AUC^PK^ and *t*_1/2_ than those of *CYP2C19* hetEMs. *CYP2C19* hetEMs shows a significantly higher C_max_ and AUC^PK^ compared to *CYP2C19* homEMs. As the previous study (Kodaira et al., 2009) revealed, no statistical significant differences were observed among *ABCB1* genotypes.

### Pharmacodynamic data

**Figure 2:**
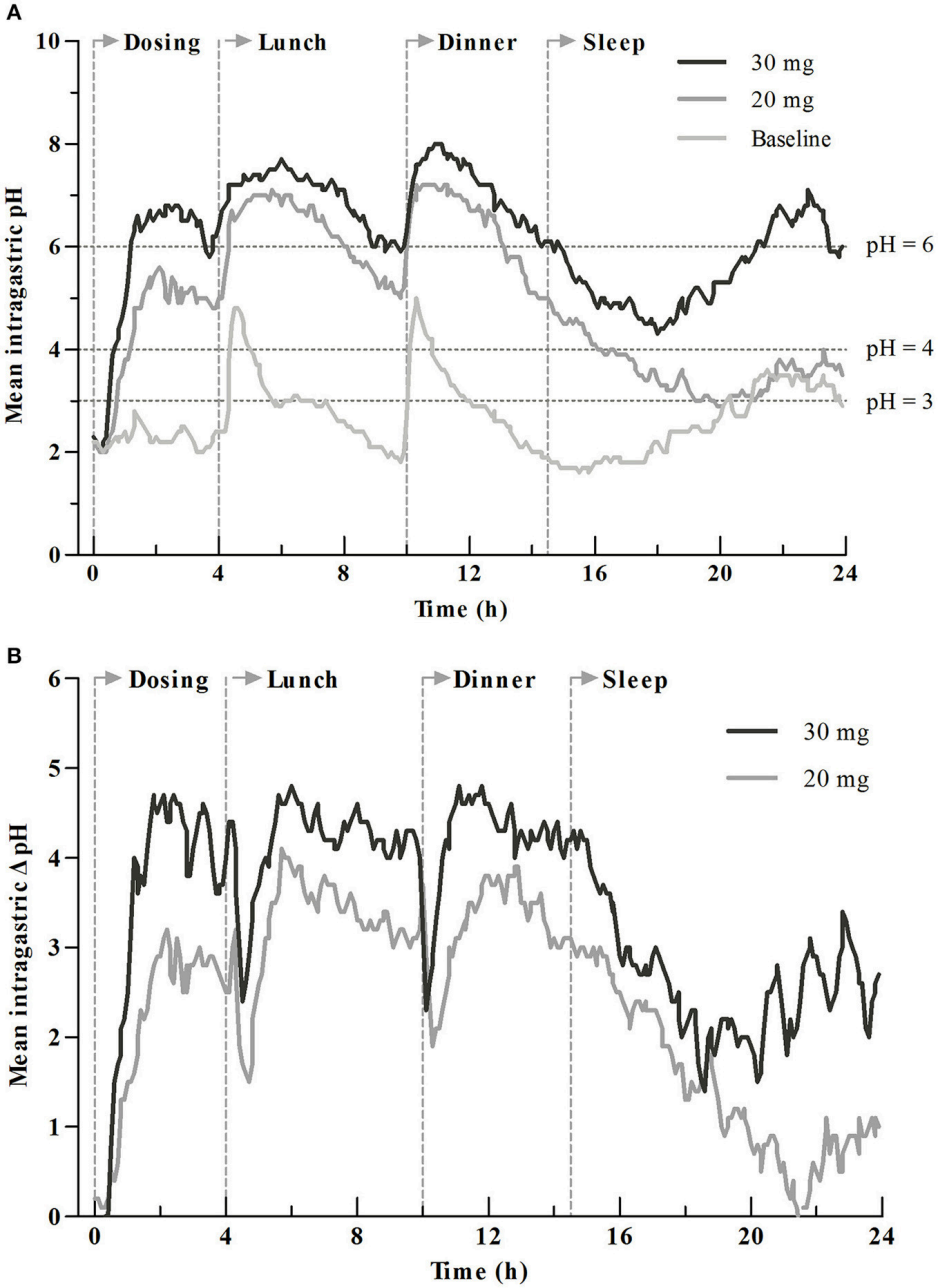
Mean intragastric pH profiles for dexlansoprazole 20 and 30 mg. **(A)** Comparison of intragastric pH before and after drug administration. **(B)** Comparison of baseline-adjusted intragastric pH by dosage. Time to first gastric pH ≥ 6 (T_pH≥6_) was 76 ± 29 min for 20 mg group and 58 ± 25 min for 30 mg group. Vertical lines at 0, 4, 10, and 14.5 h represent the time for intravenous dosing, lunch, dinner, and sleep, respectively.

The 24-h intragastric pH-time curves before and after intravenous dosing are presented in Figure 2A. The rapidity of onset of increase in intragastric pH was dose-dependent with the time to first gastric pH ≥ 6 (T_pH≥6_) of 76 (± 29) min for the 20 mg group and 58 (± 25) min for the 30 mg group. The mean intragastric pH, pH ≥ 4 and pH ≥ 6 holding times (T_pH≥4_ (%), T_pH≥6_ (%), the percentage of time that intragastric pH was above 4 or 6) for the 30 mg group was obviously higher than the 20 mg group. The baseline-adjusted intragastric pH-time curves (post-dose pH minus the baseline value) are shown in Figure 2B, and the baseline-adjusted intragastric pH was dose-dependent.

The PD parameters are summarized in Table 3. The PD parameters after intravenous administration of 20 or 30 mg dexlansoprazole were significant increased (*P* < 0.05) compared to baseline. There was a significant elevation in PD parameters in the 30 mg group than the 20 mg group during initial time (0–4 h), overnight hours (14–24 h), and the whole time (0–24 h) (*P* < 0.05) after intravenous dosing at about 8:00 a.m. Mean and median intragastric pH for the 30 mg group was significantly higher than those of the 20 mg group during 0–4, 14–24, and 0–24 h after dosing. A similar increment was observed in delta(Δ)-values (values after dosing minus the corresponding values at baseline) of mean and median intragastric pH from 20 to 30 mg. No significant differences in mean and median pH were noticed between 20 and 30 mg during the 4–10 h interval, except that Δ-mean pH for the 30 mg group was higher than that of the 20 mg group (4.2 ± 1.2 vs. 3.3 ± 1.4, *P* = 0.043). Statistically significant differences in T_pH≥3_ (%) and T_pH≥4_ (%) were observed during time intervals of 0–24, 0–4, and 14–24 h. Pairwise comparisons of baseline-adjusted T_pH≥3_ (%) and T_pH≥4_ (%) were significantly greater for 30 mg compared with 20 mg during 0–24, 0–4, and 14–24 h intervals. Significant differences were observed in both T_pH≥6_ (%) and its Δ-values between dosage groups during 0–24, 0–4, 4–10, and 14–24 h intervals. Statistically significant higher values of novel PD parameter ${\text{AUC}}_{\tau \;1-\tau \;2}^{\text{pH}-\text{t}}$ (area under pH-time curve from τ1 to τ2) were obtained with the 30 mg group compared with the 20 mg group during 0–24, 0–4, and 14–24 h intervals. Corresponding Δ-values for ${\text{AUC}}_{0-\text{24 h}}^{\text{pH}-\text{t}}$, ${\text{AUC}}_{0-4\text{h}}^{\text{pH}-\text{t}}$, ${\text{AUC}}_{4-10\text{h}}^{\text{pH}-\text{t}}$, and ${\text{AUC}}_{14-\text{24 h}}^{\text{pH}-\text{t}}$ were significantly higher with the 30 mg group vs. the 20 mg group. No statistically significant difference of above-mentioned PD parameters and Δ-values was observed between the 20 and 30 mg groups during 10–14 h post-dosing.

**Table 3:** PD parameters after single intravenous administration of dexlansoprazole 20 or 30 mg (Mean ± SD).

**Parameter**	**Dosage (mg)**	**0–24 h**	**0–4 h**	**4–10 h**	**10–14 h**	**14–24 h**
Median (pH)	20	4.9 ± 1.5^*^	4.6 ± 2.1^*^	6.3 ± 1.6	6.6 ± 1.6	3.2 ± 1.4^*^
	30	6.4 ± 1.4	6.1 ± 2.0	7.1 ± 0.9	7.3 ± 1.3	5.3 ± 1.9
ΔMedian (pH)	20	3.1 ± 1.5^*^	2.5 ± 1.9^*^	3.6 ± 1.7	3.7 ± 1.5	1.5 ± 1.3^*^
	30	4.3 ± 1.3	4.1 ± 1.9	4.6 ± 1.2	4.5 ± 1.7	3.1 ± 1.9
Mean (pH)	20	4.9 ± 1.2^*^	4.3 ± 1.5^*^	6.2 ± 1.4	6.5 ± 1.6	3.6 ± 1.3^*^
	30	6.1 ± 1.2	5.5 ± 1.5	6.9 ± 1.0	7.2 ± 1.2	5.5 ± 1.5
ΔMean (pH)	20	2.4 ± 1.0^*^	2.1 ± 1.1^*^	3.3 ± 1.4	3.3 ± 1.4	1.6 ± 1.1^*^
	30	3.4 ± 1.1	3.3 ± 1.1	4.2 ± 1.2	4.2 ± 1.5	2.8 ± 1.4
T_pH≥3_(%)	20	68.5 ± 20.9^*^	57.5 ± 22.1^*^	89.4 ± 23.1	92.7 ± 18.1	48.7 ± 30.8^*^
	30	85.1 ± 15.7	72.3 ± 20.8	96.8 ± 7.6	96.8 ± 9.5	78.9 ± 27.7
ΔT_pH≥3_(%)	20	44.5 ± 19.0^*^	47.1 ± 20.6	51.8 ± 29.6	43.8 ± 25.3	38.8 ± 27.8^*^
	30	58.6 ± 18.5	57.5 ± 22.2	60.4 ± 21.3	54.1 ± 28.5	60.0 ± 26.0
T_pH≥4_(%)	20	60.4 ± 22.2^*^	46.5 ± 26.3^*^	89.0 ± 22.1	88.4 ± 22.7	37.6 ± 31.3^*^
	30	79.8 ± 19.0	66.5 ± 23.4	94.5 ± 11.9	95.0 ± 12.9	70.6 ± 31.1
ΔT_pH≥4_ (%)	20	45.0 ± 20.7^*^	39.0 ± 22.3^*^	65.8 ± 26.9	55.5 ± 26.0	31.2 ± 28.2^*^
	30	62.8 ± 20.3	56.6 ± 19.6	75.7 ± 19.2	70.6 ± 26.4	54.8 ± 28.3
T_pH≥6_(%)	20	40.1 ± 21.3^*^	33.2 ± 29.4^*^	64.1 ± 31.0^*^	70.9 ± 35.5	16.3 ± 19.3^*^
	30	64.2 ± 21.0	54.6 ± 29.2	83.2 ± 22.3	84.5 ± 24.3	49.2 ± 25.2
ΔT_pH≥6_(%)	20	33.7 ± 18.8^*^	27.6 ± 24.2^*^	54.9 ± 32.2^*^	63.7 ± 34.3	12.0 ± 14.0^*^
	30	56.6 ± 18.8	50.7 ± 26.1	80.3 ± 22.3	82.1 ± 23.6	35.2 ± 25.6
${\text{AUC}}_{\tau \;1-\tau \;2}^{\text{pH}-\text{t}}$ (pH·h)	20	7, 079 ± 1, 725^*^	1, 043 ± 349^*^	2, 240 ± 503	1, 577 ± 378	2, 218 ± 778^*^
	30	8, 907 ± 1, 659	1, 337 ± 358	2, 498 ± 351	1, 739 ± 286	3, 332 ± 909
${\text{AUC}}_{\tau \;1-\tau \;2}^{\text{pH}-\text{t}}$ (pH·h)	20	3, 382 ± 1, 499^*^	501 ± 268^*^	1, 171 ± 519^*^	784 ± 346	942 ± 632^*^
	30	4, 934 ± 1, 578	793 ± 268	1, 498 ± 417	1, 005 ± 365	1, 638 ± 823

* *P < 0.05 20 mg vs. 30 mg. PD, pharmacodynamic; T_pH≥3_ (%), percentage time of pH ≥ 3; T_pH≥4_ (%), percentage time of pH ≥ 4; T_pH≥6_ (%), percent time of pH ≥ 6; ${\text{AUC}}_{\tau \text{1}-\tau \text{2}}^{\text{pH}-\text{t}}$, area under pH-time curve*.

**Figure 3:**
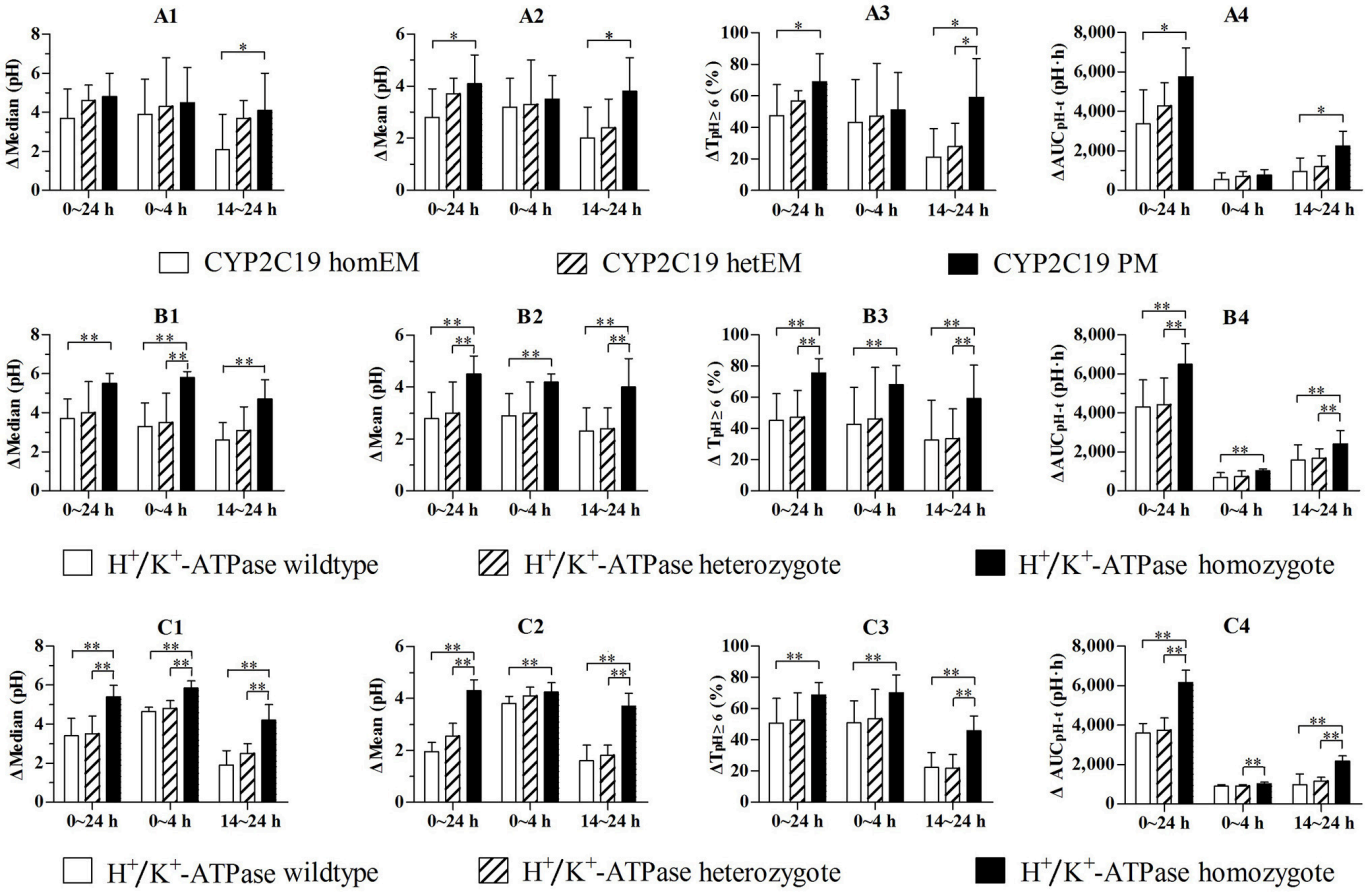
**(A1–A4)** Comparison of baseline-adjusted PD parameters by *CYP2C19* phenotype following single intravenous administration of 30 mg (*n* = 7, 10, 11 for *CYP2C19* wildtype, heterozygote, and homozygote). **(B1–B4)** Comparison of baseline-adjusted PD parameters of subjects in the 30 mg group by *H*^+^*/K*^+^*-ATPase* genotype (*n* = 8, 10, 10 for *H*^+^*/K*^+^*-ATPase* wildtype, heterozygote, and homozygote). **(C1–C4)** Comparison of baseline-adjusted PD parameters of *CYP2C19* EMs by *H*^+^*/K*^+^*-ATPase* genotype (*n* = 6, 8, 7 for *H*^+^*, K*^+^*-ATPase* wildtype, heterozygote, and homozygote). ^*^P < 0.05 vs. *CYP2C19* PM, ^**^P < 0.05 vs. *H*^+^*/K*^+^*-ATPase* homozygote.

PD parameters divided by *CYP2C19* phenotype in the 30 mg group are shown in Figures 3A1–A4, and mean values are summarized in Table 4. No significant differences in PD parameters were noticed between *CYP2C19* homEMs, hetEMs, and PMs across all time periods, except that T_pH≥6_ (%) was higher with PMs than homEMs during the 14–24 h interval. However, analysis in Δ-values for mean (pH), median (pH), T_pH≥3_ (%), T_pH≥4_ (%), T_pH≥6_ (%), and ${\text{AUC}}_{\tau \;1-\tau \;2}^{\text{pH}-\text{t}}$ showed statistically significant variations between homEMs and PMs during the 0–24 and 14–24 h intervals. Statistically significant difference between hetEMs and homEMs were found in Δ-T_pH≥6_ (%) during the 14–24 h interval additionally.

**Table 4:** PD parameters by *CYP2C19* phenotype after single intravenous administration of dexlansoprazole 30 mg (Mean ± SD).

**Parameter**	**Phenotype**	**0–24 h**	**0–4 h**	**14–24 h**
Median (pH)	EM (*n* = 11)	5.9 ± 1.7	6.2 ± 2.1	4.5 ± 2.0
	IM (*n* = 10)	7.0 ± 0.8	6.2 ± 2.8	6.2 ± 1.2
	PM (*n* = 7)	6.7 ± 1.2	5.9 ± 1.8	5.9 ± 2.0
ΔMedian (pH)	EM (*n* = 11)	3.7 ± 1.5	3.9 ± 1.8	2.1 ± 1.8^c^
	IM (*n* = 10)	4.9 ± 0.8	4.3 ± 2.5	3.7 ± 0.9
	PM (*n* = 7)	4.8 ± 1.2	4.2 ± 1.8	4.1 ± 1.9^a^
Mean (pH)	EM (*n* = 11)	5.7 ± 1.3	5.7 ± 1.6	4.9 ± 1.7
	IM (*n* = 10)	6.6 ± 0.9	5.6 ± 2.2	6.1 ± 0.9
	PM (*n* = 7)	6.5 ± 1.1	5.2 ± 1.1	6.1 ± 1.4
ΔMean (pH)	EM (*n* = 11)	2.8 ± 1.1^c^	3.2 ± 1.1	2.0 ± 1.2^c^
	IM (*n* = 10)	3.7 ± 0.6	3.3 ± 1.7	2.7 ± 0.9
	PM (*n* = 7)	4.1 ± 1.1^a^	3.5 ± 0.9	3.8 ± 1.3^a^
T_pH≥3_(%)	EM (*n* = 11)	80.0 ± 19.5	73.9 ± 21.7	67.5 ± 34.5
	IM (*n* = 10)	89.6 ± 9.2	66.9 ± 33.4	88.9 ± 8.9
	PM (*n* = 7)	89.0 ± 13.3	73.7 ± 10.5	87.3 ± 22.7
ΔT_pH≥3_(%)	EM (*n* = 11)	47.9 ± 20.2^c^	48.6 ± 24.5	47.0 ± 27.6^c^
	IM (*n* = 10)	65.3 ± 6.2	56.9 ± 27.1	60.8 ± 19.5
	PM (*n* = 7)	68.6 ± 15.3^a^	69.8 ± 9.5	76.9 ± 20.0^a^
T_pH≥4_(%)	EM (*n* = 11)	73.0 ± 23.2	69.3 ± 24.3	56.3 ± 37.2
	IM (*n* = 10)	85.7 ± 10.6	63.6 ± 36.2	80.9 ± 12.6
	PM (*n* = 7)	85.0 ± 16.4	64.8 ± 15.2	82.9 ± 26.0
ΔT_pH≥4_ (%)	EM (*n* = 11)	50.4 ± 21.3^c^	51.9 ± 17.7	38.6 ± 27.9^c^
	IM (*n* = 10)	70.0 ± 8.3	56.2 ± 31.5	55.6 ± 17.3
	PM (*n* = 7)	74.6 ± 16.6^a^	63.2 ± 13.7	75.9 ± 22.4^a^
T_pH≥6_(%)	EM (*n* = 11)	56.4 ± 24.1	58.7 ± 31.9	37.1 ± 25.0^c^
	IM (*n* = 10)	67.8 ± 13.4	56.6 ± 36.6	50.7 ± 13.6
	PM (*n* = 7)	72.2 ± 19.8	47.7 ± 24.1	64.4 ± 25.6^a^
ΔT_pH≥6_(%)	EM (*n* = 11)	47.4 ± 19.7^c^	53.0 ± 27.4	21.1 ± 18.0^c^
	IM (*n* = 10)	56.7 ± 6.4	51.3 ± 33.5	27.8 ± 14.7^c^
	PM (*n* = 7)	68.8 ± 17.9^a^	47.3 ± 23.7	59.0 ± 24.6^a^^,^^b^
${\text{AUC}}_{\tau \;1-\tau \;2}^{\text{pH}-\text{t}}$ (pH·h)	EM (*n* = 11)	8, 296 ± 1, 835	1, 376 ± 373	2, 951 ± 1, 006
	IM (*n* = 10)	9, 473 ± 1, 259	1, 354 ± 516	3, 665 ± 574
	PM (*n* = 7)	9, 343 ± 1, 617	1, 274 ± 271	3, 618 ± 881
${\text{AUC}}_{\tau \;1-\tau \;2}^{\text{pH}-\text{t}}$ (pH·h)	EM (*n* = 11)	4, 048 ± 1, 510^c^	762 ± 250	1, 188 ± 696^c^
	IM (*n* = 10)	5, 305 ± 840	789 ± 405	1, 604 ± 525
	PM (*n* = 7)	5, 867 ± 1, 562^a^	837 ± 235	2, 259 ± 818^a^

a P < 0.05 vs. EM;

b P < 0.05 vs. IM;

c *P < 0.05 vs. PM. PD, pharmacodynamic; WT, wildtype; het, heterozygote; hom, homozygote; T_pH≥3_(%), percentage time of pH ≥ 3; T_pH≥4_ (%), percentage time of pH ≥ 4; T_pH≥6_ (%), percent time of pH ≥ 6; ${\text{AUC}}_{\tau \text{1}-\tau \text{2}}^{\text{pH}-\text{t}}$, area under pH-time curve*.

PD parameters divided by gastric *H*^+^*/K*^+^*-ATPase* rs2733743 wildtypes, heterozygotes and homozygotes in the 30 mg group are shown in Figures 3B1–B4, and mean values are summarized in Table 5. No significant differences in PD parameters were noticed between gastric *H*^+^*/K*^+^*-ATPase* homEMs, hetEMs, and PMs across all time periods. However, analysis in Δ-values for mean (pH), median (pH), T_pH≥3_ (%), T_pH≥4_ (%), T_pH≥6_ (%), and ${\text{AUC}}_{\tau \;1-\tau \;2}^{\text{pH}-\text{t}}$ showed statistically significant variation between homEMs and PMs during 0–24, 0–4, and 14–24 h intervals. Statistically significant differences between hetEMs and homEMs were found in Δ-median pH, Δ-mean pH, Δ-T_pH≥6_ (%), and Δ- ${\text{AUC}}_{4-10\text{ h}}^{\text{pH}-\text{t}}$ during 0–24, 14–24, or 0–4 h intervals, respectively.

**Table 5:** PD parameters by *H*^+^*/K*^+^*-ATPase* genotype after single intravenous administration of dexlansoprazole 30 mg (Mean ± SD).

**Parameter**	**Genotype**	**0–24 h**	**0–4 h**	**14–24 h**
Median (pH)	WT (*n* = 8)	6.0 ± 1.1	5.6 ± 2.0	4.7 ± 1.7
	Het (*n* = 10)	6.0 ± 1.9	5.5 ± 2.5	4.9 ± 2.3
	Hom (*n* = 10)	7.5 ± 0.6	7.5 ± 0.6	6.7 ± 1.1
ΔMedian (pH)	WT (*n* = 8)	3.7 ± 1.0^c^	3.5 ± 1.7^c^	2.8 ± 1.7
	Het (*n* = 10)	4.0 ± 1.6	3.3 ± 2.0^c^	2.1 ± 1.9^c^
	Hom (*n* = 10)	5.5 ± 0.5^a^	5.8 ± 0.3^a^^,^^b^	4.7 ± 1.0^b^
Mean (pH)	WT (*n* = 8)	5.7 ± 0.9	5.1 ± 1.4	5.1 ± 1.3
	Het (*n* = 10)	5.9 ± 1.5	5.3 ± 2.0	5.1 ± 1.9
	Hom (*n* = 10)	7.0 ± 0.6	6.2 ± 0.8	6.6 ± 0.8
ΔMean (pH)	WT (*n* = 8)	3.0 ± 1.0^c^	2.9 ± 1.0^c^	2.6 ± 1.3
	Het (*n* = 10)	3.0 ± 1.0^c^	3.0 ± 1.2	1.9 ± 0.9^c^
	Hom (*n* = 10)	4.5 ± 0.7^a^^,^^b^	4.2 ± 0.3^a^	4.0 ± 1.1^b^
T_pH≥3_(%)	WT (*n* = 8)	81.1 ± 14.2	69.5 ± 24.9	70.7 ± 27.8
	Het (*n* = 10)	81.0 ± 20.9	70.6 ± 25.1	72.0 ± 33.7
	Hom (*n* = 10)	95.7 ± 1.3	78.0 ± 7.4	98.5 ± 1.8
ΔT_pH≥3_(%)	WT (*n* = 8)	52.6 ± 21.0^c^	54.0 ± 28.3	56.6 ± 27.3^c^
	Het (*n* = 10)	51.8 ± 14.2^c^	52.6 ± 22.2	44.1 ± 21.3^c^
	Hom (*n* = 10)	75.4 ± 7.6^a^^,^^b^	68.4 ± 8.6	84.0 ± 9.1^a^^,^^b^
T_pH≥4_(%)	WT (*n* = 8)	75.6 ± 17.1	63.3 ± 26.8	62.5 ± 31.5
	Het (*n* = 10)	74.9 ± 25.7	64.8 ± 28.6	63.2 ± 38.0
	Hom (*n* = 10)	91.7 ± 4.9	73.1 ± 12.6	91.0 ± 10.4
ΔT_pH≥4_ (%)	WT (*n* = 8)	57.3 ± 22.3	53.6 ± 23.3	50.7 ± 30.9^c^
	Het (*n* = 10)	54.9 ± 19.1^c^	51.0 ± 20.6	37.7 ± 20.5^c^
	Hom (*n* = 10)	79.9 ± 6.6^b^	67.5 ± 8.7	80.9 ± 11.2^a^^,^^b^
T_pH≥6_(%)	WT (*n* = 8)	57.1 ± 18.8	46.8 ± 27.3	43.7 ± 25.6
	Het (*n* = 10)	59.2 ± 25.9	50.5 ± 38.4	41.0 ± 26.2
	Hom (*n* = 10)	80.2 ± 7.5	70.3 ± 14.7	66.9 ± 17.6
ΔT_pH≥6_ (%)	WT (*n* = 8)	51.2 ± 17.1^c^	42.6 ± 23.6	33.3 ± 25.5^c^
	Het (*n* = 10)	47.2 ± 17.0^c^	46.0 ± 33.2	17.4 ± 11.2^c^
	Hom (*n* = 10)	75.3 ± 9.3^a^^,^^b^	67.9 ± 12.4	59.2 ± 21.4^a^^,^^b^
${\text{AUC}}_{\tau \;1-\tau \;2}^{\text{pH}-\text{t}}$ (pH·h)	WT (*n* = 8)	8, 329 ± 1, 266	1, 243 ± 339	3, 112 ± 804
	Het (*n* = 10)	8, 527 ± 2, 169	1, 296 ± 472	3, 069 ± 1, 139
	Hom (*n* = 10)	10, 171 ± 786	1, 517 ± 184	3, 955 ± 497
${\text{AUC}}_{\tau \;1-\tau \;2}^{\text{pH}-\text{t}}$ (pH·h)	WT (*n* = 8)	4, 354 ± 1, 385^c^	681 ± 248^c^	1, 572 ±776^c^
	Het (*n* = 10)	4, 311 ± 1, 380^c^	727 ± 297	1, 074 ± 478^c^
	Hom (*n* = 10)	6, 493 ± 1, 056^a^^,^^b^	1, 028 ± 84^a^	2, 405 ± 684^a^^,^^b^

a P < 0.05 vs. WT;

b P < 0.05 vs. hetrozygote;

c *P < 0.05 vs. homozygote*.

*PD, pharmacodynamic; WT, wildtype; het, heterozygote; hom, homozygote; T_pH≥3_(%), percentage time of pH ≥ 3; T_pH≥4_ (%), percentage time of pH ≥ 4; T_pH≥6_ (%), percent time of pH ≥ 6; ${\text{AUC}}_{\tau \text{1}-\tau \text{2}}^{\text{pH}-\text{t}}$, area under pH-time curve*.

PD parameters divided by gastric *H*^+^*/K*^+^*-ATPase* rs2733743 wildtypes, heterozygotes, and homozygotes based on *CYP2C19* EMs in the 30 mg group are shown in Figures 3C1–C4, and mean values are summarized in Table 6. No significant differences in PD parameters were noticed between gastric *H*^+^*/K*^+^*-ATPase* homEMs, hetEMs, and PMs across all time periods, except that T_pH≥4_ (%) was higher with homEMs than hetEMs during 0–4 h interval. However, analysis in Δ-values for mean (pH), median (pH), T_pH≥3_ (%), T_pH≥4_ (%), T_pH≥6_ (%), and ${\text{AUC}}_{\tau \;1-\tau \;2}^{\text{pH}-\text{t}}$ showed statistically significant higher values in gastric *H*^+^*/K*^+^*-ATPase* homEMs than those in PMs during 0–24, 0–4, and 14–24 h intervals. Statistically significant differences between hetEMs and homEMs were found in Δ-median pH, Δ-mean pH, Δ-T_pH≥6_ (%), and Δ- ${\text{AUC}}_{4-10\text{ h}}^{\text{pH}-\text{t}}$ during 0–24, 14–24, or 0–4 h intervals, respectively.

**Table 6:** PD parameters of *CYP2C19* EMs by *H*^+^*/K*^+^*-ATPase* genotype after single intravenous administration of dexlansoprazole 30 mg (Mean ± SD).

**Parameter**	**Genotype**	**0–24 h**	**0–4 h**	**14–24 h**
Median (pH)	WT (*n* = 6)	5.8 ± 1.4	6.9 ± 0.5	4.3 ± 1.7
	Het (*n* = 8)	4.9 ± 2.1	4.5 ± 2.8	3.5 ± 2.2
	Hom (*n* = 7)	7.5 ± 0.4	7.8 ± 0.1	6.3 ± 1.3
ΔMedian (pH)	WT (*n* = 6)	3.2 ± 1.0	4.5 ± 0.7^b^	2.3 ± 1.7
	Het (*n* = 8)	2.9 ± 1.6	1.9 ± 1.0^a^^,^^c^	0.5 ± 0.5^c^
	Hom (*n* = 7)	5.4 ± 0.3	5.9 ± 0.4^b^	4.2 ± 0.8^b^
Mean (pH)	WT (*n* = 6)	5.6 ± 1.1	6.1 ± 0.5	4.8 ± 1.8
	Het (*n* = 8)	5.1 ± 1.7	4.7 ± 2.4	4.1 ± 2.1
	Hom (*n* = 7)	6.8 ± 0.2	6.6 ± 0.5	6.2 ± 0.4
ΔMean (pH)	WT (*n* = 6)	2.4 ± 0.8^c^	3.4 ± 0.7	1.6 ± 0.9^c^
	Het (*n* = 8)	2.3 ± 0.6^c^	2.2 ± 0.8^c^	1.2 ± 0.4^c^
	Hom (*n* = 7)	4.3 ± 0.4^a^^,^^b^	4.3 ± 0.4^b^	3.7 ± 0.5^a^^,^^b^
T_pH≥3_(%)	WT (*n* = 6)	79.8 ± 16.2	80.8 ± 8.4	62.4 ± 35.6
	Het (*n* = 8)	70.1 ± 26.3	63.7 ± 35.7	53.0 ± 41.0
	Hom (*n* = 7)	95.1 ± 1.1	78.7 ± 10.0	96.8 ± 1.2
ΔT_pH≥3_ (%)	WT (*n* = 6)	35.3 ± 10.3^c^	51.7 ± 34.6	38.5 ± 26.5^c^
	Het (*n* = 8)	40.8 ± 11.1^c^	36.4 ± 20.1	31.5 ± 13.0^c^
	Hom (*n* = 7)	77.4 ± 6.3^a^^,^^b^	62.5 ± 11.6	83.1 ± 5.9^a^^,^^b^
T_pH≥4_(%)	WT (*n* = 6)	73.1 ± 20.0	76.4 ± 9.4	52.1 ± 41.3
	Het (*n* = 8)	62.9 ± 33.1	56.9 ± 39.3^c^	44.6 ± 48.0
	Hom (*n* = 7)	87.9 ± 4.7	77.1 ± 11.6^b^	80.4 ± 6.2
ΔT_pH≥4_ (%)	WT (*n* = 6)	41.0 ± 16.6^c^	58.6 ± 15.0	29.5 ± 29.9
	Het (*n* = 8)	41.4 ± 17.3^c^	35.0 ± 11.7	25.1 ± 16.9
	Hom (*n* = 7)	78.0 ± 1.5^a^^,^^b^	67.2 ± 2.4	72.4 ± 1.5
T_pH≥6_(%)	WT (*n* = 6)	53.5 ± 23.6	66.9 ± 14.8	34.4 ± 28.0
	Het (*n* = 8)	48.0 ± 32.5	39.4 ± 48.2	30.2 ± 32.6
	Hom (*n* = 7)	73.4 ± 3.7	75.5 ± 12.2	51.5 ± 10.9
ΔT_pH≥6_ (%)	WT (*n* = 6)	43.2 ± 18.2	60.9 ± 12.6	14.1 ± 12.6^c^
	Het (*n* = 8)	37.3 ± 19.1	33.8 ± 39.0	11.6 ± 2.9^c^
	Hom (*n* = 7)	68.7 ± 7.9	70.1 ± 4.5	45.8 ± 17.4^a^^,^^b^
${\text{AUC}}_{\tau \;1-\tau \;2}^{\text{pH}-\text{t}}$ (pH·h)	WT (*n* = 6)	8, 178 ± 1, 587	1, 462 ± 111	2, 900 ± 1, 050
	Het (*n* = 8)	7, 409 ± 2, 391	1, 147 ± 577	2, 505 ± 1, 248
	Hom (*n* = 7)	9, 806 ± 345	1, 588 ± 124	3, 697 ± 221
${\text{AUC}}_{\tau \;1-\tau \;2}^{\text{pH}-\text{t}}$ (pH·h)	WT (*n* = 6)	3, 372 ± 1, 064^c^	817 ± 157	968 ± 553^c^
	Het (*n* = 8)	3, 317 ± 850^c^	531 ± 194^c^	755 ± 198^c^
	Hom (*n* = 7)	6, 157 ± 629^a^^,^^b^	1, 024 ± 83^b^	2, 168 ± 270^a^^,^^b^

a P < 0.05 vs. WT;

b P < 0.05 vs. hetrozygote;

c *P < 0.05 vs. homozygote. PD, pharmacodynamic; EM, extensive metabolizer; WT, wildtype; het, heterozygote; hom, homozygote; T_pH≥3_(%), percentage time of pH ≥ 3; T_pH≥4_ (%), percentage time of pH ≥ 4; T_pH≥6_ (%), percent time of pH ≥ 6; ${\text{AUC}}_{\tau \text{1}-\tau \text{2}}^{\text{pH}-\text{t}}$, area under pH-time curve*.

## Discussion

PPIs are acid-liable, so they are usually prepared as enteric-coated formulation to protect from degradation in the stomach when given orally (Horn and Howden, 2005). Oral administrated PPIs in humans are absorbed in the proximal small intestine and suffered from many factors, including preparation disintegrate and release, gastrointestinal motility rate, and the first-pass metabolism (Vanderhoff and Tahboub, 2002; Horn and Howden, 2005; Zhang et al., 2011; Chai et al., 2017). The pharmacokinetics of PPIs after oral administration, compared with IV administration, are affected by the dosage form, food and drink, gastrointestinal physiological conditions, and other various factors (Vanderhoff and Tahboub, 2002; Horn and Howden, 2005; Zhang et al., 2011; Chai et al., 2017). Interindividual variation of PK parameters after oral administration were larger than that after IV route (Amer et al., 2004; Freston et al., 2004; Thota et al., 2013). In order to decrease the influence of drug absorption and the first-pass metabolism on the inhibition of acid secretion by PPIs, the intravenous infusion was superior to others. Dexlansoprazole was selected to conduct the study to reduce the influence of the stereoselective PK of enantiomers (Katsuki et al., 1996; Metz et al., 2009; Sun et al., 2015).

The interindividual variation of PK parameters of lansoprazole and dexlansoprazole by *CYP2C19* phenotype were smaller than that of the un-phenotype group (Qiao et al., 2006; Xu et al., 2010; Li et al., 2014). Moreover, the interindividual variation of PK parameters by *CYP2C19* phenotype after IV administration were smaller than that after oral administration. To minimize the impact of metabolic phenotypes of *CYP2C19*, intravenous infusion of dexlansoprazole injection in *CYP2C19* EMs population were optimized to elucidate the relation between SNPs of *H*^+^*/K*^+^*-ATPase* and the inhibition of acid secretion.

The gastric acid inhibition of PPIs depends on their irreversible covalent binding to gastric H^+^*/K*^+^-ATPase, leading to a much longer duration of suppression than its plasma half-life (Sachs et al., 2007). Our results showed remarkably higher intragastric pH after dosing in all subjects when compared to their own baseline, particularly during the nocturnal time. The elevation in intragastric pH was dose-dependent and the higher the dose the greater the increments of gastric pH inhibition were observed. So far, whether the efficacy of PPIs is affected by gastric *H*^+^*/K*^+^*-ATPase* polymorphism in humans has not been clearly established. Our results reveal that gastric *H*^+^*/K*^+^*-ATPase* rs2733743 homozygotes have significantly stronger gastric acid inhibition than wildtypes after administration of dexlansoprazole injection.

Main PK parameters (such as C_max_, AUC^PK^, and half-life) of dexlansoprazole were higher than the data of lansoprazole injection at the same dosage (Yang et al., 2012). Results of the current study show that *t*_1/2_ and AUC^PK^ for *CYP2C19* PMs were about 3- to 4-fold of those for *CYP2C19* homEMs in the same dose group, which was consistent with previous studies of PK performance affected by *CYP2C19* polymorphism after oral and intravenous administration of lansoprazole (Hu et al., 2004; Wang et al., 2012). There were statistically significant differences of baseline-adjusted mean pH (Δ ${\text{AUC}}_{\tau \;1-\tau \;2}^{\text{pH}-\text{t}}$) and T_pH≥6_ (%) between PMs and homEMs during the 0–24 or 14–24 h intervals. The stronger and longer acid inhibition in PMs could be explained by much higher C_max_ and AUC^PK^ caused by a slower clearance and a higher system exposure. Lansoprazole is also a substrate of *ABCB1*, but the significant effects of *ABCB1* polymorphism on pharmacokinetics and pharmacodynamics of dexlansoprazole have not been found in this study.

Further analysis comparing the pharmacodynamic parameters between gastric *H*^+^*/K*^+^*-ATPase* and *CYP2C19* genotypes in the dexlansoprazole 30 mg group was aiming to reveal which genotype would suppress gastric acid secretion more strongly. The results showed that there was no significant difference between *CYP2C19* homEMs, hetEMs, and PMs during the first 4 h after intravenous administration of dexlansoprazole, while gastric *H*^+^*/K*^+^*-ATPase* homozygotes had significantly higher gastric acid inhibition effects of Δ ${\text{AUC}}_{\tau \;1-\tau \;2}^{\text{pH}-\text{t}}$, T_pH≥3_(%), and T_pH≥4_ (%) than gastric *H*^+^*/K*^+^*-ATPase* heterozygotes or wildtypes. And in the *CYP2C19* EMs, we also get the same results in that the subjects classified as gastric *H*^+^*/K*^+^*-ATPase* rs2733743 homozygotes had significantly higher gastric acid inhibition effects than those classified as *H*^+^*/K*^+^*-ATPase* heterozygotes or wildtypes.

In the summary, we propose that gastric *H*^+^*/K*^+^*-ATPase* rs2733743 genotypes have greater effects than *CYP2C19* genotypes on the suppression of gastric acid secretion. In the meanwhile, we could explain why some patients could not get satisfactory gastric acid inhibition after a conventional dose of PPIs.

## Conclusion

Gastric *H*^+^*/K*^+^*-ATPase* polymorphism affects intragastric pH-values after PPIs dosing, which may cause insufficient gastric acid inhibition. Gastric *H*^+^*/K*^+^*-ATPase* genotypes had greater effects than *CYP2C19* genotypes on the suppression of gastric acid secretion. A single dose of 30 mg dexlansoprazole injection provides more effective acid inhibition compared to 20 mg.

## Author contributions

Each of the authors participated in this research by contributing to the conception and design of the study (YW and YC), study management (YW, LS, YL, HZ, MW, LX, JC, MB, HL, PPZ, XZ, and PZ), performance of laboratory experiments (LS, YF, and ZY), and statistical analysis and interpretation (YW, LS, YL, JW, and LM).

### Conflict of interest statement

The authors declare that the research was conducted in the absence of any commercial or financial relationships that could be construed as a potential conflict of interest.
